# Radiomics and Digital Image Texture Analysis in Oncology (Review)

**DOI:** 10.17691/stm2021.13.2.11

**Published:** 2021-01-01

**Authors:** A.A. Litvin, D.A. Burkin, A.A. Kropinov, F.N. Paramzin

**Affiliations:** Professor, Department of Surgical Disciplines, Immanuel Kant Baltic Federal University, 14 A. Nevskogo St., Kaliningrad, 236016, Russia; Deputy Head Physician for Medical Aspects, Regional Clinical Hospital of the Kaliningrad Region, 74 Klinicheskaya St., Kaliningrad, 236016, Russia; PhD Student in Information Science and Computer Engineering, Immanuel Kant Baltic Federal University, 14 A. Nevskogo St., Kaliningrad, 236016, Russia; Therapeutist, Central City Clinical Hospital, 3 Letnyaya St., Kaliningrad, 236005, Russia; Oncologist, Central City Clinical Hospital, 3 Letnyaya St., Kaliningrad, 236005, Russia

**Keywords:** radiomics, analysis of tissue textures, image biomarkers, quantitative analysis of digital images, digital image analysis in oncology, virtual biopsy

## Abstract

One of the most promising areas of diagnosis and prognosis of diseases is radiomics, a science combining radiology, mathematical modeling, and deep machine learning. The main concept of radiomics is image biomarkers (IBMs), the parameters characterizing various pathological changes and calculated based on the analysis of digital image texture. IBMs are used for quantitative assessment of digital imaging results (CT, MRI, ultrasound, PET). The use of IBMs in the form of “virtual biopsy” is of particular relevance in oncology.

The article provides the basic concepts of radiomics identifying the main stages of obtaining IBMs: data collection and preprocessing, tumor segmentation, data detection and extraction, modeling, statistical processing, and data validation. The authors have analyzed the possibilities of using IBMs in oncology, describing the currently known features and advantages of using radiomics and image texture analysis in the diagnosis and prognosis of cancer. The limitations and problems associated with the use of radiomics data are considered.

Although the novel effective tool for performing virtual biopsy of human tissue is at the development stage, quite a few projects have already been implemented, and medical software packages for radiomics analysis of digital images have been created.

## Introduction

Early diagnosis of malignant tumors determines the success of treatment and improves the prognosis of the disease. Various medical imaging modalities are used for this purpose: ultrasound, X-ray computed and magnetic resonance imaging (CT and MRI), positron emission tomography (PET), single-photon emission computed tomography (SPECT), as well as hybrid methods — PET/CT, PET/SPECT, PET/MRI. Digital images of the tumor obtained with their aid reflect its anatomical and functional changes. However, most of these data are largely nonspecific and insufficiently informative [1–3].

Radiomics, a new direction for in-depth digital image analysis, has been rapidly developing in recent years [4]. The annual increase in the number of published papers on this subject has been 177.8% (p<0.001) [5].

The concept of radiomics was first proposed in 2012 [6]. This science involves high-throughput extraction, analysis, and interpretation of quantitative features from medical images [5–7]. Texture analysis of images is part of radiomics and provides an objective quantitative assessment of tumor heterogeneity by distributing and interconnecting the levels of pixels or grayscale voxels in an image [8, 9]. Given the non-invasiveness of the radiomic method, texture analysis of images can be presented as a “virtual biopsy” [10]. The goal of radiomics and texture analysis is to build a standardized prognostic model to determine clinical outcomes with selected features. The main diagnostic task of radiomics in oncology is accurate differentiation between benign and malignant tumors using non-invasive diagnostic methods [11–13].

**The aim of this review** is to analyze papers devoted to the features of radiomics, which are currently being developed or already used in clinical practice, and textural analysis of medical images, making it possible to carry out non-invasive diagnosis of various oncological diseases.

## Literature search method

A systematic literature search was carried out in the PubMed database using the search line “Radiomics” [All Fields] AND “digital image texture analysis” [All Fields] and eLibrary — “radiomics” and “digital image texture analysis”. The search interval was 2016–2020. All published works devoted to the use of radiomics and digital image texture analysis in medicine and oncology were studied.

## Basic terms of radiomics

Radiomics is a hybrid analytical process aimed at determining the correlation between the characteristics of a digital image of tissues (including tumor tissues) and involves the following steps: data collection and preprocessing, tumor segmentation, data detection and extraction, modeling, statistical processing, and data validation [14, 15].

The radiomics workflow begins with image acquisition. Then the study region (a given region of interest) is processed using special software. Furthermore, certain parameters (functions, indicators) — image biomarkers (IBMs) — are selected in the processed statistical model. Work on images includes various stages of preliminary and subsequent processing [16–18].

In the statistical model, the first step is to estimate the frequency distribution of the gray level based on a histogram of pixel intensity in the given region of interest, including the average intensity, threshold (percentage of pixels in the specified range), entropy (randomness), standard deviation, skewness, and kurtosis (peak/ flatness of the histograms of pixels). Second-order statistics involves such parameters as second-order entropy, energy, homogeneity, difference, and correlation.

Higher-order statistics — contrast, “coarseness”, and “occupancy” — can be calculated using grayscale difference matrices that study the location and relationship between three or more pixels/voxels. The statistical model is also checked [19]. Data collection relies on a large number of medical images and related clinical data to reveal the existing correlation between them [17, 18].

The details of obtaining IBMs for radiomics analysis are described in numerous studies of the effectiveness of digital imaging: CT [20–23], PET [24–27], MRI [28–30], ultrasound [31–33]. Although the technical aspects of image preprocessing and filtering have been well developed to date [34–37], the efficacy of these functions in predicting the course of the disease is being intensively studied [38–42].

The software implementation of radiomics analysis of digital images is based on both commercial software solutions and open-source programs [43, 44]. These programs tend to generate a large number of texture features, many of which are common to all software, but not all studies use the same descriptors, making it difficult to compare the results. Besides, the same name of a texture feature can sometimes cover different calculation methods or different names of characteristics [45]. Today, there have been developed such commercial packages as RADIOMICS™ (OncoRadiomics, the Netherlands) and TexRAD™ (Feedback Medical Ltd., Great Britain) [45]. There are also non-commercial open-source software platforms — LIFEx [43], IBEX (Imaging Biomarker Explorer) [44], Pyradiomics [46]. The most interesting is the IBEX tool, which evaluates five main indicators: the gray level co-occurrence matrix, the gray-level run-length matrix, the neighborhood intensity difference matrix, histogram, and shape [44, 47, 48]. Work is underway to standardize IBMs, which will create the standardized terminology of image processing workflow and provide guidelines for conducting research in the field of radiomics [49, 50].

Segmentation determines which region will be analyzed (region of interest — ROI) and includes manual, semi-automatic, and automatic methods. Manual segmentation is an important step in the radiomics workflow, as radiological features are extracted from segmented regions of interest [51]. Automatic or semi-automatic segmentation techniques are widely studied to minimize manual input and improve consistency in delineating regions of interest [52]. However, today there are no proven common standards for tumor segmentation and its implementation is time-consuming [51, 52]. There are many variations in morphological features since tumors are very different from geometric objects. Tumor margins can be “blurred” because they are unclearly defined in most medical images [53].

The essence of radiomics is creating mathematical models and algorithms that receive medical images at the input of computer analysis and give out the pathophysiological features of tissues as the output [54, 55]. To create such a model, it is necessary to go through several stages [56, 57]. The first stage (formulation of a clinical task) is identifying the tumor phenotype to select the optimal therapeutic approach, estimating the susceptibility to a particular drug, or predicting the likelihood of side effects from therapy. The second stage is collecting a database of medical images relevant to the task at hand. The third stage is data markup [58, 34]. Then, for each selected region of all selected images, IBMs are calculated. The following IBMs are distinguished:

shape features: volume, maximum linear size, area, compactness, and sphericity, the interrelationship between these characteristics;

first-order features (histogram features describe the statistical properties of pixels in the selected region of the image): the maximum, minimum, mean, and median values of the intensity in the selected region, standard deviation from the mean, skewness of the distribution;

second-order features: textural features of the correlation of neighboring pixel values and the homogeneity of the selected region [58].

Higher-order features describe the statistical characteristics of images obtained from the original ones by applying various mathematical methods: Fourier transform, wavelet analysis, as well as various filters [34].

Image biomarkers obtained by machine learning methods are selected by algorithms automatically. The most popular algorithms in radiomics are regression, various types of decision trees, and neural networks [59–61]. The most informative IBMs are selected from the entire set of calculated features using mathematical statistics [62, 63]. Removing the uninformative features makes the prediction results more stable and prevents random “noise” in the data from influencing the decision. A mathematical model is built (trained) based on the obtained features, which predicts the necessary features — tumor phenotype, susceptibility to the chosen treatment modality, the likelihood of side effects, etc. [64, 65].

## The use of radiomics in clinical practice

Radiomics lies at the intersection of radiology, computer science, and mathematical statistics. Medical images contain information inaccessible to the naked eye. This hidden information can be extracted by applying a series of mathematical transformations to the resulting images. The results of these transformations can correlate with pathophysiological properties invisible in the images. Knowledge of the pathophysiological properties makes it possible to get a better understanding of the disease details in each specific case and to choose the optimal treatment modality [66, 67]. Radiomics is the most promising for the diagnosis and treatment of cancer. For example, its methods make it possible to determine the phenotype of a malignant tumor without resorting to the invasive procedure of biopsy and to select drugs with the highest efficacy [68]. In fact, radiomics can reveal the microscopic parameters of the investigated tissues from macroscopic images of the investigated object [67].

Providing a more accurate non-invasive diagnosis, radiomics analysis has come into use as a way to predict the overall survival of cancer patients. Wang et al. [69] investigated the informativeness of radiomics based on the analysis of 411 CT scans of patients with locally advanced rectal cancer who received neoadjuvant chemotherapy followed by surgery. The authors determined the values of radiomics features that allow dividing patients into low-risk and high-risk survival groups. Bae et al. [70] studied the role of the method in improving survival prognosis in patients diagnosed with glioblastoma multiforme. They extracted IBMs from 217 multivariate MRI scans and identified 18 of the most informative radiomics characteristics that can significantly improve patient stratification when considered in addition to clinical and genetic profiles. Oikonomou et al. [71] studied IBMs from 150 PET/CT scans of patients receiving stereotactic radiation therapy for lung cancer. They constructed signatures using 10 functions and found radiomics analysis to be a good predictor of overall survival. Kirienko et al. [72] extracted IBMs from PET, CT, and combined PET/CT images of patients with non-small cell lung cancer after surgery (n=295) and developed radiomics signatures predicting relapse-free survival in this category of patients.

There have been studied the possibilities of radiomics in non-invasive differentiation of histological subtypes of non-small cell lung cancer. For example, Wu et al. [73] extracted IBMs from 350 CT scans of patients with adenocarcinoma and squamous cell lung cancer, whose tumor histology was determined on surgical specimens. The authors developed a signature of five radiomics features with fairly good diagnostic characteristics — AUC=0.72.

Filatau et al. [74] used the capabilities of CT imaging biomarkers for the differential diagnosis of chronic pancreatitis and pancreatic cancer. According to the authors, the overall accuracy of differential diagnosis (the accuracy of the method) was 0.92.

Wu et al. [75] obtained IBMs from 170 MRI scans of patients with hepatocellular carcinoma. The histological characteristics of the tumors were established using remote surgical specimens. The signature of radiomics only (AUC=0.74) outperformed the clinical model (AUC=0.60), while their combination significantly improved the prediction of the grade of hepatocellular carcinoma — AUC=0.80. Vallèries et al. [76] achieved a sensitivity of 0.96 and a specificity of 0.93 in the diagnosis of metastatic lung lesions using models with combined IBMs based on PET and MRI.

The effectiveness of IBMs has been confirmed for predicting the immune response to therapy in oncological diseases. A radiomics signature has been developed that predicts the response to immunotherapy in patients with advanced melanoma and patients with non-small cell lung cancer (AUC=0.76) [77]. The role of IBMs was studied in assessing the complete clinical response after neoadjuvant chemoradiation therapy in patients with locally advanced rectal cancer. The IBMs obtained from 114 MRI images produced a radiomics signature with a sensitivity of 1.0 and a specificity of 0.91 that surpassed the qualitative assessment of the analysis performed by two radiologists [78].

Automatic segmentation of target structures can be performed using radiomics tools [79]. Jiang et al. [80] developed a model of three-dimensional segmentation of a lung tumor on CT images. It was trained using examination results of 377 patients from an open-access dataset available from The Cancer Imaging Archive (https://www.cancerimagingarchive.net). For validation, two independent datasets were used, consisting of examination results in 304 and 529 patients with lung tumors. Interestingly, there was no significant difference between the mask generated by their model and the manual segmentation by the experts.

Manual segmentation of brain glioblastoma in MRI is a very time-consuming process. An automated model for brain tumor segmentation developed by Yi et al. [81] based on 274 MRI images extracted from an open-access dataset [82] can greatly facilitate the process. The model accuracy is 0.89.

Chen et al. [83] proposed a model capable of detecting and segmenting cervical tumors using PET imaging; its accuracy is 0.84.

Besides, IBMs can be used for tumor classification tasks. Ardila et al. [84] studied the possibility of predicting the risk of lung cancer by means of screening low-dose CT. The authors trained their model on 7000 images and tested its effectiveness on 1139 cases (AUC=0.94). Interestingly, the predictions made by the model were more accurate than those of radiologists (n=6). This contributed to a significant reduction in the number of false positive (11%) and false negative (5%) results.

Abdelaziz Ismael et al. [85] investigated the possibility of using radiomics algorithms for differential diagnosis of various brain tumors. The authors developed an algorithm based on 3064 MRI images from 233 patients. The classification accuracy was 0.99 (based on MRI data only) and 0.97 (on clinical testing). Sibille et al. [86] used a combination of CT  +  PET to differentiate lung masses in 629 patients with cancer or lymphoma. The algorithm developed by the authors demonstrated high accuracy (AUC=0.98).

Radiomics features have also been studied to assess the response to cancer therapy. The potential of IBMs for predicting the response to radiation therapy in patients with lung cancer (primary or metastatic) has been reported with an accuracy of 0.72 [87]. There was proposed an algorithm that achieved a sensitivity of 0.81 and a specificity of 0.82 in predicting the response to neoadjuvant chemotherapy in patients with esophageal cancer based on PET scanning [88].

Image biomarkers are good diagnostic and prognostic tools in oncology, capable of improving the prognosis of distant metastases [89], pathological response to treatment [90], local recurrence [91], sensitivity to chemoradiotherapy [92], relapse-free survival [93], radiation pneumonitis, etc. [94].

However, despite the first encouraging results, there are limitations to the use of radiomics and digital image texture analysis in oncology and medicine in general. The main limitation of the wide use of radiomics is the fact that the type of tissue texture analysis performed, the type of segmentation used, post-processing methods, and the quantity and quality of texture object output vary widely across platforms and studies, making comparison of results difficult. At the moment, there are no unified standards for measuring radiomics parameters and tissue texture. Despite statistically significant results, there is a wide variation in the published data [5].

The next major problem with radiomics is the enormous amount of data obtained from texture analysis of medical images. Moreover, the study of several features on the same dataset can lead to a significant probability of error and generation of false results [95]. When analyzing a large number of IBMs, the values must be adjusted to test multiple hypotheses [96, 97]. In addition to the above-mentioned factors, there are other limitations, such as metallic artifacts in CT images [98], the peak voltage and current of the CT X-ray tube [99], and others that also might affect the quantitative assessment of radiomics features.

Taking into account the influence of various imaging parameters, researchers should pay more attention to standardizing imaging protocols and provide the necessary parameters to achieve reproducibility and comparability with other radiometric studies [100].

## Conclusion

Radiomics and tissue texture analysis in digital imaging is a new area of medical research that allows non-invasive virtual biopsy of human tissue. Particularly relevant is the modern quantitative analysis of tissue characteristics using image biomarkers in oncology, which allows improving the results of diagnosis, differentiation of tumors, as well as making decisions on treatment strategy and predicting outcomes. Advances in data mining and machine learning make it possible to extract many quantitative features and transform the fast-growing number of medical images into data required by clinical oncologists.
